# Supplementary material for: Identification of photoperiod-induced specific miRNAs in the adrenal glands of Sunite sheep (Ovis aries)
Source: Front Vet Sci. 2022 Jul 22;9:888207. doi: 10.3389/fvets.2022.888207 (PMC9354845; doi:10.3389/fvets.2022.888207)
Supplement: Supplementary file 1 [file Data_Sheet_1.ZIP › Supplementary file/miRDeep2 file.zip!/miRDeep2 file/Novel_Structure/SPLP42A2/Novel_546.pdf]

Provisional ID	: NC_019484.2_43651
Score total	: 8.8
Score for star read(s)	: 3.9
Score for read counts	: 1.7
Score for mfe	: 1.6
Score for randfold	: 1.6
Score for cons. seed	:
Total read count	: 15
Mature read count	: 9
Loop read count	: 0
Star read count	: 6

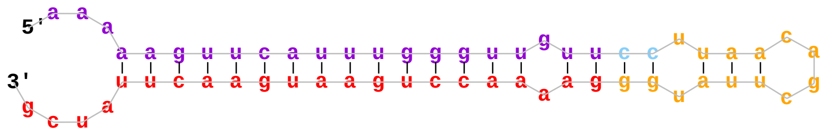

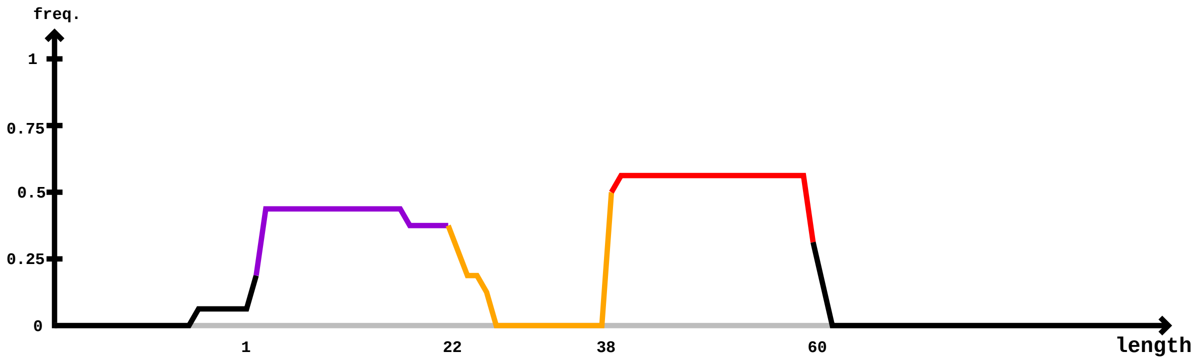

Star Mature

```
5'- uacuacuauuguguuggccaaaaaguucauuuggguuguuccuuaacagcuuaugggaaaaccugaaugaacuuaucggucaacccaauacguuagagaaauguagagugacag   -3'   obs
    uacuacuauuguguuggccaaaaaguucauuuggguuguuccuuaacagcuuaugggaaaaccugaaugaacuuaucggucaacccaauacguuagagaaauguagagugacag         exp
    ..(((((((((.(((((((...((((((((((((((.((((.(((....))).)))).)))))))))))))))...))))))).))))).).)))....................   reads   mm   sample
    .............uuggGcaaaaaguucauuuggg...............................................................................   1       1    seq
    ...................aaaaaguucauuuggguuguu..........................................................................   2       0    seq
    ....................aaaaguucauuuggguuguuG.........................................................................   1       1    seq
    ....................aaaaguucauuuggguuguuccA.......................................................................   1       1    seq
    ....................aaaaguucauuuggguuguuccuu......................................................................   2       0    seq
    ........................................................gaaaaccugaaugaacuuauc.....................................   4       0    seq
    ........................................................gaaaaccugaaugaacuuaucg....................................   4       0    seq
    .........................................................aaaaccugaaugaacuuaucg....................................   1       0    seq
```
